# Liquid Biopsy–Based Profiling of *TP53* Gene Hotspot Mutations in HBV‐Induced Chronic Liver Disease and Hepatocellular Carcinoma Patients: A Study From Bangladesh

**DOI:** 10.1155/ijh/6175143

**Published:** 2026-08-17

**Authors:** Sabinur Akter, Sharmin Sultana, Md. Zulqarnine Ibne Noman, Farzana Laila, Afzalun Nessa

**Affiliations:** ^1^ Department of Virology, Bangladesh Medical University (BMU), Dhaka, Bangladesh; ^2^ Department of Microbiology, Shaheed Mansur Ali Medical College & Hospital, Dhaka, Bangladesh; ^3^ Programme of Emerging Infections (PEI), Infectious Disease Division (IDD), icddr, b, Dhaka, Bangladesh, icddrb.org

**Keywords:** Bangladesh, cell-free DNA, Hepatitis B virus, hepatocellular carcinoma, liquid biopsy, mutation, *TP53* gene

## Abstract

**Background:**

The tumor protein (*TP53*) gene is a common driver gene for somatic mutations in hepatocellular carcinoma(HCC), and detection may provide insight into evaluating disease progression. Circulating cell‐free DNA (cfDNA) from liquid biopsy has emerged as a promising noninvasive biopsy material for identifying potential genetic alterations. This study explored the mutational signature of the *TP53* gene in Hepatitis B virus (HBV)–infected chronic liver disease (CLD) and HCC patients from cfDNA.

**Method:**

A total of 80 participants: 30 HCC, 30 CLD, and 20 healthy controls were included in this cross‐sectional study. cfDNA was extracted from aseptically collected peripheral venous blood. *TP53* (R249S) gene was amplified using selected primers via conventional PCR and visualized on a 2% agarose gel electrophoresis. Amplified products underwent Sanger sequencing; mutational analysis was done using MEGA11 software compared with the reference sequence.

**Result:**

*TP53* hotspot mutations were found in 23.3% (7/30) of HCC patients. Mutational frequencies were detected: 13.3% (4/30) at position 746G > A (R249S), 6.7% (2/30) at 747G > A (R249S), and 3.3% (1/30) at 735C > T (G245S). Among CLD patients, a single hotspot mutation (3.3%) was detected at position 744G > A/T(R248Q/W). However, no hotspot mutation was detected in healthy controls. All HCC patients with mutations (7/7, 100%) had elevated ALT, AST, serum bilirubin, and AFP levels, and in the unmutated (wild) group, these were 69.6%, 47.8%, 30.4%, and 60.9%, respectively. The mean AST and AFP levels were significantly higher in mutated patients compared with the wild group.

**Conclusion:**

Detection of *TP53* gene mutations along with biochemical markers in the management of HCC is evident, and it may play an important role in targeted therapy and personalized treatment plans.

## 1. Introduction

Hepatocellular carcinoma (HCC) forms more than 80% of all primary liver cancers, and the burden is increasing daily [1]. Globally, around 44% of liver cancer cases are linked to Hepatitis B virus (HBV) [2]. An estimated 254 million incident cases of HBV and about 1.1 million people died in 2022 from HBV‐related complications [3]. Bangladesh is one of the high‐burden countries that have a significant number of cases of HCC, ranking as the third most prevalent cancer [4].

About 90% of hepatocytes have been found to have integrated HBV DNA at multiple chromosomal sites in HBV‐induced HCC patients [5]. This integration can cause genetic instability, uncontrolled cell proliferation, and malignant transformation. The top 5 mutant genes in HCC patients reported from different geographical locations are *TP53*, *TERT*, *CTNNB1*, *AXIN1*, and *ARID2* [6].

The *TP53* gene, often called the “guardian of the genome,” encodes the tumor protein p53, a key tumor suppressor that controls cell growth and division [7]. p53 acts as a crucial checkpoint during cell division [8]. When DNA damage occurs due to factors like radiation, toxins, or ultraviolet (UV) light, p53 either halts the cell cycle to allow repair or triggers apoptosis if the damage is irreparable. Mutations in *TP53* disrupt this protective function, allowing abnormal cells to divide unchecked, which can lead to tumor formation and cancer progression [9]. Mutations in the *TP53* gene most commonly occur in Exons 5–7; approximately 30% of all mutations in the *TP53* gene are concentrated at six hotspot codons, including R175H, G245S, R248Q, R248W, R249S, R273C, R273H, and R282W. These hotspot mutations are located primarily within the DNA‐binding domain of the p53 protein, which is crucial for its tumor‐suppressive function. Alterations at these sites often lead to loss of DNA‐binding ability and gain of oncogenic properties, contributing to cancer progression and poor clinical outcomes [10].

Although great progress has been made in chronic liver disease (CLD) and HCC therapy in recent decades, about 25%–70% of patients are identified at advanced stages when available therapeutic methods are ineffective [11]. Liver biopsy is currently a method used for detection of any somatic gene alteration as well as for diagnosis of advanced stage and invasiveness of the tumor [12]. The risk of biopsy includes sampling error, pain, bleeding, and even shedding of tumor along the needle track. However, these shortcomings can be overcome by analyzing liquid biopsy methods. Liquid biopsy generally refers to the analysis of blood or other body fluids to obtain genetic or epigenetic information. Cell‐free DNA (cfDNA) from blood plasma samples, referred to as liquid biopsy, can be an effective tool in the early detection of any somatic mutations in affected cells for many cancers such as HCC, melanoma, lung cancer, and breast cancer [13].

Bangladesh lacks sufficient data on mutations in the *TP53* gene in cases of chronic hepatitis and HCC patients, as well as the use of cfDNA as an alternative to invasive tissue biopsy. To address this gap, the study was designed to detect the presence of hotspot mutations in the *TP53* gene among patients with HBV‐induced chronic hepatitis and HCC.

## 2. Methods

### 2.1. Study Participant Selection

This was a cross‐sectional study conducted among 80 participants, of whom 30 had HCC, 30 had CLD, and 20 were apparently healthy controls. The duration of the study was 1 year, from January to December 2024, and participants were recruited from the Hepatology In‐Patient and Out‐Patient Department of Bangladesh Medical University (BMU) on the basis of selection criteria. For the HCC patient group, HBV‐induced HCC cases confirmed by a hepatologist, evidenced by histopathology and/or imaging reports (CT scan, MRI, histopathology, and FibroScan), and with serological and biochemical markers of HBV infection within the last 2 months were included. For the chronic Hepatitis B patient group, serological evidence of chronic HBV infection (HBsAg positive for > 6 months) was considered, and for the healthy control group, apparently healthy individuals with negative results for HBsAg and total anti‐HBc, no history of CLD or any other chronic disease, and no history of acute liver disease within the last 6 months were included. Patients with HCC/CLD other than HBV, secondary metastatic deposition in the liver and other cancers, any other primary liver cancer (caused by HCV, NASH, and alcoholism), HBV‐induced HCC/CLD with HCV and/or any other cancer, and those in a moribund condition, thus unable to provide information, were excluded from this study. The study proposal was approved by the Institutional Review Board of BMU (*BSMMU/2024/6106*). Informed written consent was obtained from each participant. The study followed a purposive sampling method to recruit the participants. Face‐to‐face interviews were conducted and recorded in a predesigned data collection sheet. Results of relevant current and previous investigations were taken from their medical records.

#### 2.1.1. Sample Size

The sample size was calculated using a two‐proportion *Z*‐test formula (performed using Stata 17), aiming to detect a difference in the prevalence of mutations between the HCC and CLD groups. Based on prior clinical observations, the expected mutation prevalence was 31.25% in the HCC group and 6.60% in the CLD group. With a two‐sided significance level (*α*) set at 0.05 and a study power of 70%, the required sample size was calculated to be 30 participants per group. Additionally, 20 age‐ and sex‐matched healthy controls were recruited. As the primary objective was to compare the mutation prevalence between the two disease groups (HCC vs. CLD), the healthy control group served as a baseline reference to confirm the absence of the mutation in the general population. Considering the logistical challenges and ethical limitations of recruiting healthy volunteers in a single‐center setting, a 3:2 case‐to‐control ratio was maintained for the healthy control cohort.

### 2.2. Blood Sample Collection and cfDNA Extraction

Peripheral venous blood was collected from the antecubital vein of each participant with all aseptic precautions and kept in a properly labeled EDTA‐containing vacutainer, centrifuged at 4000 rpm for 10 min at room temperature, and plasma was carefully transferred into new plain red tubes within 30 min of sample collection and then stored, maintaining a gradual freezing cycle. cfDNA was extracted using the QIAamp Circulating Nucleic Acid Extraction Kit (Qiagen, Germany, Catalog No.: 55114), following the manufacturer′s protocol. At first, all the frozen samples were allowed to thaw at room temperature before proceeding to cfDNA extraction. The isolated circulating cfDNA was measured using a microvolume UV spectrophotometer. The ratio of absorbance at 260 and 280 nm and the absorbance spectrum were used to assess the purity of DNA. DNA samples with a 260/280 ratio between 1.8 and 2.0 were considered to have maximal purity. The mean cfDNA concentration among the HCC, CLD, and healthy control groups was 39.4, 28.2, and 21.8, respectively (Table S1).

### 2.3. PCR Amplification of *TP53* Gene

Conventional PCR was performed using the AgileCycler (Infinigen Biotech Inc., United States) instrument. Amplification of extracted circulating cfDNA was done using the selected primers: sense 5 ^′^‐CTTGCCACAGGTCTCCCCAA‐3 ^′^ and antisense 5 ^′^‐AGGGGTCAGCGGCAAGCAGA‐3 ^′^ [14]. PCR amplification was carried out in a 25 *μ*L volume containing GoTaq G2 Green Master Mix (Promega, United States), 1 *μ*L forward and reverse primer each, 5 *μ*L genomic cfDNA, 12.5 *μ*L green master mix, and nuclease‐free water to make each reaction in separate reaction tubes. PCR conditions were set with an initial denaturation at 95°C for 2 min, followed by 40 cycles of denaturation at 95°C for 30 s, annealing at 60°C for 45 s, elongation at 72°C for 30 s, and final elongation at 72°C for 10 min.

### 2.4. Gel Electrophoresis of Amplified Product

**Figure 1:**
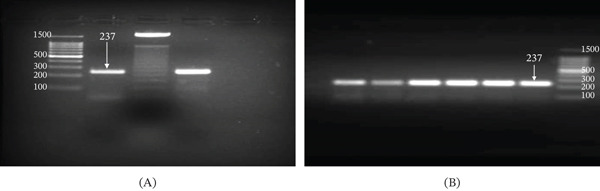
(A) Gel documentation of the PCR products from 02 specimens of the selected region of the *TP53* gene with the DNA ladder on a 2% agarose gel (first and third lanes: ladder; second and fourth lanes: amplified product). (B) Gel documentation of the PCR products from 06 specimens of the selected region of the *TP53* gene with a DNA ladder on a 2% agarose gel (first to sixth lanes: amplified product; seventh lane: ladder).

PCR‐amplified product size was 237 bp, and it was confirmed by gel electrophoresis on a 2% agarose gel along with a 1500 bp DNA ladder (ABclonal) as a marker. PCR amplicon of each sample was stored at −20°C until further use (Figure 1).

### 2.5. Nucleotide Sequencing of Amplified Gene Products

The nucleotide sequencing was carried out on the automated ABI 3500Xl Genetic Analyzer (Applied Biosystems, Foster City, United States). Cycle sequencing was done using BigDye Terminator v3.1 Cycle Sequencing Kit containing ready reaction premix (RRP). After performing cycle sequencing, DNA products were purified using the BigDye XTerminator Purification Kit. The bottle of BigDye XTerminator beads was vortexed for 8–10 s before mixing with SAM solution. After mixing with the purification reagents, the reaction mixture was vortexed for 30 min at 1800 rpm. After that, the cleaned‐up sequencing reaction mixture was transferred to a MicroAmp Optical 96‐Well Reaction Plate, and the plate was sealed with septa. Finally, the plate was placed for a short spin‐down before proceeding to capillary electrophoresis. Capillary electrophoresis was done in an ABI 3500 Genetic Analyzer (Applied Biosystems, United States). BDx_Std_Seq_50_POP7_Z module was used for the run. Generated chromatograms were saved for further analysis.

### 2.6. Statistical and Bioinformatics Analysis

**Figure 2:**
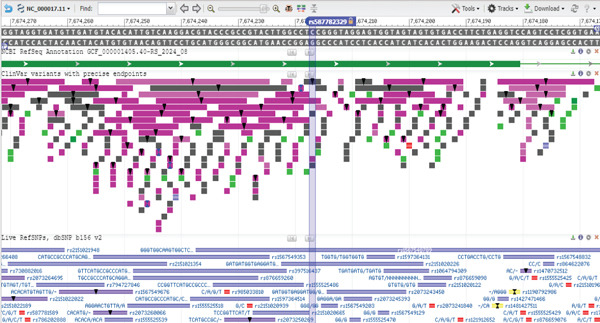
*TP53* gene sequence from the NCBI gene database, indicating 746 hotspot regions. Here, the *TP53*‐selected portion of the gene sequence was identified using the rsID number. For *TP53* hotspot region 746, rs587782329.

**Figure 3:**
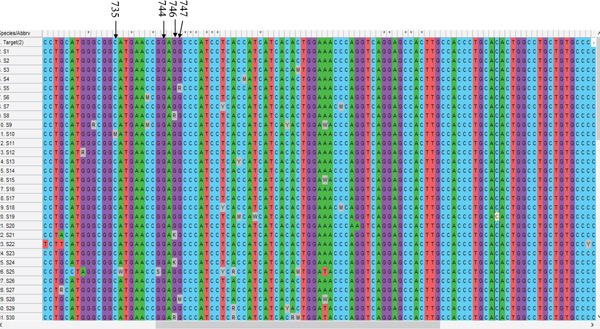
Alignment of the *TP53* gene of Exon 7 in HBV‐induced HCC patients′ hotspot (735, 746, and 747) mutations using MEGA11 software. A 735 hotspot mutation was found in Sample No. 10; 746 in Sample Nos. 8, 21, 24, and 30; and 747 in Sample Nos. 5 and 28.

Sequencing chromatograms were visualized in Finch TV software to assess the base quality. Chromatograms were visualized and edited in MEGA 11 [15] using the NCBI Nucleotide BLAST program (https://blast.ncbi.nlm.nih.gov/Blast.cgi), whereas mutation analysis was done by comparing the query sequence with the reference sequence of the *TP53* gene (Figures 2 and 3). From the survey sheet, data was compiled in Microsoft Excel 2023. Data checking, verification, and cleaning were done. Statistical analysis was performed using STATA 17 (Stata Corp LLC, United States). The demographic variables, clinical profile, and biochemical and liver markers were analyzed and presented as numbers, means, medians, standard deviations, ranges, and percentages. Categorical variables were assessed by Fisher′s exact test and chi‐square test, whereas continuous variables were analyzed by unpaired *t*‐test and Mann–Whitney *U* test. *p* value < 0.05 was considered significant.

## 3. Result

The sociodemographic profile of the study participants is presented in Table 1. The mean age of the healthy control was 39.7 ± 12.08 years, whereas in CLD and HCC patients, it was 36.3 ± 11.32 and 50.57 ± 11.78 years, respectively. Male participants were predominant (86.7% and 93.3%) in both patient groups. The majority of the patients in the HCC group (73.3%) resided in rural areas. The history of smoking was reported more frequently in the HCC group (56.7%) in comparison with the CLD group (30%) and the healthy group (15%).

**Table 1:** Sociodemographic features of study participants (*N* = 80) among the three groups.

Demographic variable name	Healthy control, *n* (%)	HBV‐induced (*n* = 60)		*p* value
		CLD, *n* (%)	HCC, *n* (%)	
Age (years)				**0.003** ^**a**^
Mean ± SD	39.7 ± 12.08	36.3 ± 11.32	50.57 ± 11.78	
Median (range)	36 (26–69)	34 (19–64)	47 (30–77)	
Gender				**< 0.001** ^**b**^
Male	10 (50.0)	26 (86.7)	28 (93.3)	
Female	10 (50.0)	4 (13.3)	2 (6.7)	
Residence				0.083
Rural	10 (50.0)	14 (46.7)	22 (73.3)	
Urban	10 (50.0)	16 (53.3)	8 (26.7)	
History of smoking	3 (15.0)	9 (30.0)	17 (56.7)	**0.016** ^**b**^

*Note:* Data are presented as mean ± standard deviation, range (min–max), frequency, and percentage. Superscript “a” indicates a *t*‐test was done, superscript “b” indicates Fisher′s exact test was done, and, in other cases, a chi‐square test was done. *p* value was calculated by analyzing the difference between the healthy and HCC groups.

A significantly higher number of HCC patients were reported with jaundice (53.3% vs. 6.7%), ascites (70% vs. 6.7%), palmar erythema (26.7% vs. 0%), portal hypertension (70% vs. 6.7%), history of variceal bleeding (16.7% vs. 0%), and edema (46.7%) in comparison with CLD patients (Table 2). Within the HCC cohort, multifocal tumor distribution was observed in 67.7% of patients with a tumor diameter exceeding 3 cm in 70% of cases (Table 2).

**Table 2:** Clinical profile of the patients with HBV‐induced CLD and HCC (*N* = 80).

Variable name	Healthy control, *n* (%)	CLD, *n* (%)	HCC, *n* (%)	*p* value^a^
Jaundice	**—**	2 (6.7)	16 (53.3)	**< 0.001**
Ascites	—	2 (6.7)	21 (70.0)	**< 0.001**
Palmar erythema	**—**	0 (0.0)	8 (26.7)	**< 0.001**
Portal hypertension	—	2 (6.7)	21 (70.0)	**< 0.001**
History of variceal bleeding	**—**	0 (0.0)	5 (16.7)	**0.012**
Edema	**—**	2 (6.7)	14 (46.7)	**< 0.001**
Tumor number				*NA*
Single	**—**	**—**	10 (33.3)	
Multiple	**—**	**—**	20 (66.7)	
Tumor size				*NA*
< 3 cm	**—**	**—**	21 (70)	
> 3 cm	**—**	**—**	9 (30)	

*Note:* Data are presented as frequency and percentage. *p* value < 0.05 is considered significant and is shown in bold.

Abbreviation: NA, not applicable.

^a^Fisher′s exact test was done.

The total *TP53* hotspot mutation frequency in HCC patients was 23.3% (7/30), whereas it was 3.3% among the CLD patient group, which was statistically significant (*p* = 0.01) (Table 3).

**Table 3:** Presence of the hotspot mutations among healthy controls and HBV‐induced CLD and HCC patients (*N* = 80).

Position of the mutation	Healthy, *n* (%)	CLD, *n* (%)	HCC, *n* (%)	*p* value^a^
*TP53* gene				
747G > T	0	0	2 (6.7)	0.34
746G > A	0	0	4 (13.3)	**0.04**
744G > A/T	0	1 (3.3)	0	1.00
735C > T	0	0	1 (3.3)	1.00
Total	0	1 (3.3)	7 (23.3)	**0.01**

*Note:* Data are presented as frequencies and percentages. *p* value < 0.05 is considered significant and is shown in bold.

Abbreviations: A, adenine; C, cytosine; G, guanine; T, thymine.

^a^Fisher′s exact test was done.

Most participants in the wild‐type and mutation groups among HCC patients were male. Males were 91.3% of the wild‐type group and 100% of the mutation group (*p* = 1.0). Similarly, the majority of participants in both groups were from rural areas (73.9% vs. 71.4%, respectively). There was no significant association between *TP53* mutation status and place of residence (*p* = 1.0). Patients with *TP53* mutations showed higher levels of ALT (≥ 45 IU/L) and AST (≥ 35 IU/L) compared with the wild‐type group. All mutation‐positive patients had elevated ALT and AST levels, whereas only 69.6% and 47.8% of wild‐type patients had elevated levels, respectively (*p* = 0.154 and *p* = 0.024). Serum bilirubin (≥ 1.2 mg/dL) was significantly associated with *TP53* mutations, as all mutation patients had elevated bilirubin levels compared with 30.4% of the wild‐type group (*p* < 0.002). A significant difference was observed between low serum albumin levels (< 3.4 g/dL) and *TP53* mutations. A total of 71.4% of mutation‐positive patients had low albumin levels, whereas 56.5% of the wild‐type group showed low albumin (*p* = 0.669). AFP levels (≥ 20 ng/mL) were significantly higher in patients with *TP53* mutations. Elevated AFP levels were observed in 100% of mutation‐positive patients, compared with 60.9% of the wild‐type group (*p* = 0.071) (Table 4).

**Table 4:** *TP53* gene hotspot mutation in relation to demographic and biochemical markers among HBV‐induced HCC patients (*N* = 30).

Variable name, *N* = 30	*TP53* gene, *n* = 30 (%)		*p* value^a^
	Wild type (*n* = 23)	Mutation (*n* = 7)	
Gender			1.0
Male	21 (91.3)	7 (100)	
Female	02 (8.7)	0 (0)	
Residence			1.0
Urban	6 (26.1)	2 (28.3)	
Rural	17 (73.9)	5 (71.4)	
ALT (SGPT)			0.154
≥ 45 IU/L	16 (69.6)	7 (100)	
< 45 IU/L	7 (30.4)	0	
AST (SGOT)			**0.024**
≥ 35 IU/L	11 (47.8)	7 (100)	
< 35 IU/L	12 (52.2)	0	
Albumin (g/L)			0.669
≥ 3.4 g/dL	10 (43.5)	2 (28.6)	
< 3.4 g/dL	13 (56.5)	5 (71.4)	
Serum bilirubin			**0.002**
≥ 1.2 mg/dL	7 (30.4)	7 (100)	
< 1.2 mg/dL	16 (69.6)	0	
AFP (ng/mL)			0.071
≥ 20 ng/mL	14 (60.9)	7 (100)	
< 20 ng/mL	9 (39.1)	0	

*Note:* Data are presented as frequency and percentage. *p* value < 0.05 is considered significant and is shown in bold.

^a^Fisher′s exact test was done.

The differences in mean ALT, albumin, and bilirubin between wild type and hotspot mutation were not significant (*p* value > 0.05), whereas the mean AST (79.5 vs. 156.8; *p* value 0.02) and the mean AFP (33.8 vs. 60.4; *p* value 0.04) between wild type and hotspot mutation were statistically significant (Table 5).

**Table 5:** Status of biochemical markers among hotspot mutations in HBV‐induced HCC patients (*N* = 30).

Name of hepatic enzyme	Mean (wild type, *n* = 23)	Mean (hotspot mutation, *n* = 7)	*p* value
ALT	92.7 (±113.2)	150.2 (±49.2)	0.21
AST	79.5 (±75.5)	156.8 (±67.3)	**0.02**
Albumin	3.7 (±1.2)	3.02 (±0.7)	0.19
Bilirubin	2.9 (±2.4)	3.1 (±3.6)	0.85
AFP	29.5 (±33.8)	60.4 (±34.2)	**0.04**

*Note:* Data are presented as mean ± standard deviation. *t*‐test was done. *p* value < 0.05 is considered significant and is shown in bold.

## 4. Discussion

cfDNA, extracted from peripheral blood, is an excellent, easily accessible liquid biopsy material that can be used for genetic study instead of invasive procedures. In recent years, liquid biopsy–based biomarkers, such as cfDNA, have shown promise for early disease detection, predicting disease progression, prognosis, molecular detection of any genetic alteration, and guiding personalized treatment [16, 17]. Although cfDNA has been extensively studied in various cancers, its role in HCC diagnosis remains less clear. Establishing cfDNA as a reliable biomarker for HCC management requires further research [18, 19]. In the present study, the mean concentration of cfDNA among HCC, CLD, and healthy control participants was 39.4, 28.2, and 21.8 ng, respectively. Post hoc pairwise comparison showed that the mean concentration was significantly higher in the HCC group compared with the CLD group (mean difference = 11.24, *p* = 0.006) (Tables S1 and S2). In previous studies, average cfDNA concentration correlates directly with both tumor size and advanced cancer stages, showing a steady increase as the disease progresses [20, 21].

In this study, a stark contrast in *TP53* mutation frequencies was observed between HCC and CLD cohorts (23.3% vs. 3.3%), aligning with global data documenting *TP53* mutation in 20%–40% of HCC cases [22, 23]. The minimal mutation rate in CLD suggests that these disruptions are late‐stage events in a stepwise hepatocarcinogenesis framework, acting as indicators of malignant transformation. This study revealed prominent Exon 7 hotspots: 747G > T, 746G > A, 744G > A/T, and 735C > T. Notably, a specific transversion mutation at *TP53* codon 249 (747G > T, pR249S) has been frequently observed in HCC patients with HBV in regions with high dietary aflatoxin B1 exposure including India [14, 22, 24]. Findings of this study reported a mutation in the same codon 249 (747G > T and 746G > T), which is also common in lung cancer and is associated with tobacco smoking. In this study, smoking history was more prevalent in HCC patients (56.7%) compared with CLD patients (30%), suggesting that smoking may contribute to gene mutation and progression of CLD to HCC. This finding is supported by previous studies that have identified smoking as an independent risk factor for HCC, particularly in the context of chronic viral hepatitis [25, 26].

Male predominance in both HCC (93.3%) and CLD (86.7%) cohorts of this study is consistent with the global epidemiology of these conditions. Males are more likely to engage in high‐risk behaviors such as smoking and alcohol consumption, which are established risk factors for liver diseases [27]. The rural–urban distribution of the study population revealed notable patterns, with a higher proportion of HCC patients (73.3%) originating from rural areas compared with CLD patients (46.7%). A relatively higher number of patients with hotspot mutations detected from rural areas may reflect differences in exposure to environmental risk factors, such as aflatoxin‐rich food intake or untreated hepatitis infections, which are more common in rural settings [28]. In rural Bangladesh, the warm, humid climate creates an ideal environment for *Aspergillus* molds to thrive. When staple foods are harvested and stored using traditional methods, they easily retain moisture. A previous study revealed that some common Bangladeshi dietary practice like dates, groundnuts, betel nuts, lentils, and red chili powder found to be contaminated with excessive levels of aflatoxin [29].

Limited access to healthcare in rural populations may delay the diagnosis and treatment of CLD, increasing the risk of progression to HCC [27]. Elevated ALT, AST, serum bilirubin, and AFP levels in all (100%) HCC patients with *TP53* gene mutations, compared with 30.4%–60.9% of unmutated HCC patients reported in this study, also indicate a more aggressive nature of the disease with poor outcomes.

The findings of this study provide valuable insights for both clinical practice and future research. Specifically, patients with *TP53* gene mutations may benefit from enhanced surveillance and individualized treatment approaches [23]. Despite the valuable insights into the rate and clinical significance of *TP53* mutations in Bangladeshi HCC and CLD patients, there were a few limitations of this study. The sample size was small that may limit the generalizability of the findings. Although this study′s cross‐sectional framework prevents us from proving a direct causal link between genetic mutations and clinical outcomes, longitudinal research is vital to better understand the temporal relationship between these mutations and the progression from CLD to HCC. This research also did not include paired tumor and peritumoral tissue samples for validating driver mutations, as the primary aim was to analyze molecular changes in circulating tumor DNA (ctDNA). Comparing the results of cfDNA with tissue samples may have increased the strength of this study.

## 5. Conclusion

Findings of this study demonstrate that *TP53* gene mutations are more frequent in Bangladeshi HCC patients compared with CLD patients and healthy controls. *TP53* gene mutations detected from liquid biopsy may be used as biomarkers, along with liver enzymes, in HCC patients for early detection, management, and prognosis of HCC. These gene mutations can also be used in precision diagnostics and targeted therapeutics as part of a personalized treatment plan.

## Author Contributions

Sabinur Akter, Sharmin Sultana, and Afzalun Nessa conceived the study idea, research design, and protocol writing. Sabinur Akter and Farzana Laila collected data. Sabinur Akter, Md. Zulqarnine Ibne Noman, and Afzalun Nessa analyzed and processed the data and interpreted the results. All authors contributed to writing the initial draft.

## Funding

This study was funded by the Bangladesh Medical University.

## Disclosure

All authors approved the final manuscript.

## Ethics Statement

This study was approved by the Institutional Review Board of BMU (*BSMMU/2024/6106*). All participants involved in the study provided written informed consent.

## Conflicts of Interest

The authors declare no conflicts of interest.

## Supporting information


**Supporting Information** Additional supporting information can be found online in the Supporting Information section. The supporting file includes additional tables (Tables S1 and S2) that provide detailed concentrations and ratios of cfDNA among healthy controls and the HBV‐induced CLD and HCC patients and supporting data related to the post hoc pairwise mean cfDNA concentration comparison among the three groups.

## Data Availability

The datasets analyzed during the current study are available from the corresponding author on reasonable request.
