# Supplementary material for: Liquid Biopsy–Based Profiling of TP53 Gene Hotspot Mutations in HBV‐Induced Chronic Liver Disease and Hepatocellular Carcinoma Patients: A Study From Bangladesh
Source: Int J Hepatol. 2026 Aug 17;2026:6175143. doi: 10.1155/ijh/6175143 (PMC13479801; doi:10.1155/ijh/6175143)
Supplement: Supplementary file 1 [file IJH-2026-6175143-s001.docx]

**Supplementary Data for Liquid Biopsy-Based Profiling of TP53 gene hotspot Mutations in HBV-induced Chronic Liver Disease and Hepatocellular Carcinoma Patients: a study from Bangladesh**

**Supplementary Table S1:** The concentration and ratio of the cfDNA among Healthy control and the HBV induced CLD and HCC patients (N=80)

| ***Group*** |  | ***Concentration (ng)*** | ***Ratio*** |
| --- | --- | --- | --- |
| ***HCC*** | *Mean (SD)* | 39.4 (16.2) | 1.8 (0.12) |
|  | *Median (IQR)* | 37.6 (26.4-43.6) | 1.8 (1.72-1.92) |
| ***CLD*** | *Mean (SD)* | 28.2 (12.1) | 1.9 (0.25) |
|  | *Median (IQR)* | 25.1 (19.5-34.6) | 1.87 (1.76-1.92) |
| ***Normal*** | *Mean (SD)* | 21.8 (18.9) | 1.9 (0.14) |
|  | *Median (IQR)* | 19.4 (9.5-29.9) | 1.9 (1.8-2.0) |

Post-hoc pairwise comparison showed that the mean concentration was significantly higher in the HCC group compared to the CLD group (mean difference = 11.24, p = 0.006). The Normal group had significantly lower mean concentration than the HCC group (mean difference = −17.62, p < 0.001). However, no statistically significant difference was observed between the Normal and CLD groups (p = 0.160) (Table-4).

**Supplementary Table S2:** Post-Hoc pair-wise mean cfDNA concentration comparison among the three groups

| ***Comparison*** | ***Mean Difference*** | ***p-value*** | ***Interpretation*** |
| --- | --- | --- | --- |
| ***HCC vs CLD*** | 11.24 | 0.006 | Significant difference |
| ***Normal vs CLD*** | -6.38 | 0.160 | Not statistically significant |
| ***Normal vs HCC*** | -17.62 | <0.001 | Significant difference |
